# Development of a biosensor for spectrophotometric determination of l-lactate in artificial saliva

**DOI:** 10.1186/s13065-025-01718-5

**Published:** 2026-01-30

**Authors:** Rehab E. Bayoumy, Nariman A. El-Ragehy, Nagiba Y. Hassan, Amr M. Mahmoud

**Affiliations:** Pharmaceutical Analytical Chemistry Department, Faculty of Pharmacy, Cairo University, Kasr-El-Aini, Cairo, 11562 Egypt

**Keywords:** Artificial saliva, Colorimetric sensors, Ferric ion, Lactate oxidase, l-Lactate, Nanozymes, Peroxidase-like activity, TMB

## Abstract

Analysis of critical biomarkers like l-lactate is extremely important in clinical practice. Herein, a non-invasive and sensitive colorimetric biosensor for accurate l-lactate determination has been developed. The proposed method demonstrates the ability of Fe^3+^ ions of iron(III) chloride to substitute the traditional horseradish peroxidase enzyme in the colorimetric determination of l-Lactate. The biosensor is based on the release of H_2_O_2_ by lactate oxidase enzyme (LOx) after 30 min incubation in a 37 °C water bath. Subsequently, H_2_O_2_ reacts with 3,3′,5,5′-tetramethylbenzidine substrate (TMB) catalyzed by Fe^3+^ ion utilizing its peroxidase-mimetic activity. Fe^3+^ ion has peroxidase-like activity which could rapidly catalyze the oxidation reaction of TMB by H_2_O_2,_ producing a characteristic blue colored product at 30 °C water bath for 15 min. Based on the catalytic mechanism of fast electron transfer between TMB and H_2_O_2_ with the assistance of the intrinsic peroxidase-like activity of Fe^3+^ ion, a colorimetric biosensor for determination of l-lactate was developed. The obtained colored product of oxidized TMB could be measured spectrophotometrically at λmax 652 nm. The biosensor yielded a reproducible response over a linear range of 5 µM–20 µM of l-lactate with a limit of detection of 1.278 µM. Furthermore, satisfactory results were obtained upon application of the method to artificial saliva samples.

**Supplementary Information:**

The online version contains supplementary material available at 10.1186/s13065-025-01718-5.

## Introduction

A biomarker, also known as a molecular marker or disease signature molecule, that can be used to determine how effectively the body reacts to a treatment for a disease or condition [1]. Biomarkers can be specific cells, molecules, or genes, gene products, enzymes, or hormones [2]. l-lactate is a normal metabolic biomarker which is produced in skeletal muscles, the brain, red blood cells, and the kidneys from pyruvate under anaerobic conditions. l-Lactate is eliminated rapidly at a rate of 320mmol/L/hr in normal human subjects primarily through liver metabolism [3]. Plasma lactate concentration reflects a complicated relationship between a variety of factors influencing the balance between lactate production and lactate clearance. Blood lactate is used as a fitness indicator, as it increases to 12mM and 25mM during exercise and extensive work respectively [4]. Moreover, l-Lactate is an important indicator in emergency medicine that shows the severity and prognosis of conditions including sepsis, lactic acidosis, trauma, cardiac arrest and reduced renal excretion [5]. The most common techniques used to determine l-lactate concentration are electrochemical methods [6], chemiluminescence [7, 8], spectrophotometric assays [9–12], HPLC [13–15] and fluorometry [16]. Biosensors are considered an exciting scientific area for research as they are highly useful in multidisciplinary fields [17–19]. In addition, the colorimetric methods are one of the most required techniques because of their ease of use, affordability, speed, and high universality [20].

Conventionally, blood or serum samples have been used to quantify l-lactate at values between 0.5 and 2.5 mM [21]. Blood samples mainly require experienced staff, also handling and stability of the samples should be considered [22]. The current direction in l-lactate measurement is focused on less stressful, non-invasive biological fluids such as sweat, tears, and other biological fluids where l-lactate could be measured, especially Saliva [23]. l-lactate can be measured in saliva as it is secreted from salivary glands and also its passive diffusion from blood. There is a high correlation between salivary lactate (SL) and capillary blood lactate (CBL) concentration which makes it a suitable candidate for non-invasive analysis especially for critical-care patients and athletes. Moreover, several inspiring works were reported for determination of l-lactate in saliva upon literature survey [23–26]. For the colorimetric quantification of l-lactate, it is generally determined using the enzyme lactate oxidase (LOx), which catalyzes the conversion of l-lactate to pyruvate and releases H_2_O_2_ as a byproduct. H_2_O_2_ and thus l-lactate determination has been accomplished using a variety of detection protocols, including electrochemical, chemiluminescence, and colorimetric detection. The combination of horseradish peroxidase (HRP) together with lactate oxidase is a well-established approach for l-lactate determination via H_2_O_2_ reduction. The usage of natural enzymes, especially using more than one simultaneously, in biosensor development has some disadvantages leading to less stable and overpriced biosensors. Researchers are trying to replace natural enzymes with artificial enzyme to overcome the problems of natural enzymes. To avoid using horseradish peroxidase (HRP) and more than one enzyme simultaneously, various nanoparticles (metal or metallic oxide) have peroxidase-like activity [27–29]. They catalyze the reaction of H_2_O_2_ with colorless peroxidase substrates such as 3,3′,5,5′-Tetramethylbenzidine (TMB) or o-phenylenediamine (OPD) to their corresponding colored products. Peroxidase-like iron oxides or iron-modified oxides have ideally been utilized owing to their catalytic properties that depend on their intrinsic peroxidase-like activities. According to Fenton reaction, iron (Fe^2+^/Fe^3+^) catalyzes the decomposition of H_2_O_2_ to generate hydroxyl radical ^⋅^OH. Hydroxyl radical can oxidize the peroxidase substrates and thus exhibit peroxidase-like activity [30–33]. In addition, these materials are more economically feasible than nanoparticles of precious metals like gold or silver. Nowadays, the use of synthesized artificial enzymes, or nanozymes, in place of natural enzymes is a real possibility due to substantial advancements in materials science and nanotechnology. However, in contrast to natural enzymes, the nanozymes usually are less catalytically active and the procedures of preparation are complicated. The stability of these nanozymes, reproducibility and building complex nanostructures are technical challenges in fabrication of convenient NPs for biologics. Herein, we developed a colorimetric biosensor for determination of l-lactate. The biosensor uses lactate oxidase enzyme and exploits the Fe^3+^ ion peroxidase mimic activity to catalyze the TMBH_2_O_2_ system to develop the color [34].The color intensity was measured spectrophotometrically at λmax 652 nm wavelength. The method was applied to artificial saliva as a non-invasive important biological matrix.

## Experimental

### Pure materials

Sodium l-lactate (with certified purity ≥ 99.0%, MW: 112.06 g/mol), hydrogen peroxide 30% and lactate oxidase (LOx) enzyme 50 IU (from *Aerococcus viridans*, 41 units/mg solid lyophilized powder) were obtained from Sigma Aldrich (St. Louis, MO, United States).

### Reagents

3,3’,5,5’-tetramethylbenzidine (TMB), ferric chloride anhydrous (≥ 99.99%), dimethyl sulfoxide (DMSO) and 2-Amino-2-(hydroxymethyl)-1,3-propanediol (trizma or tris base, ≥ 99.9%) were obtained from Sigma Aldrich (St. Louis, MO, United States). Trizma solution was adjusted to pH 8.5 with 0.1 M hydrochloric acid to obtain the buffer. Glacial acetic acid and sodium acetate of analytical grade were purchased from El Nasr Pharmaceutical Chemicals Co. (Egypt). Ultra-pure water (Millipore) was used throughout the study. LOx lyophilized powder (50 IU) is reconstituted immediately before use in 1 mL ultra-pure water.

### Artificial saliva

The chemical composition of artificial saliva (pH 7.2) included (3.90 mM Na_3_PO_4_, 4.29 mM NaCl, 17.98 mM KCl, 1.10 mM CaCl_2_, 0.08 mM MgCl_2_, 0.50 mM H_2_SO_4_, 3.27 mM NaHCO_3_ all dissolved in water) [35].

### Instruments

Shimadzu 1650 UV–VIS double-beam spectrophotometer (Kyoto, Japan) was used for the absorption spectral measurements.

### Procedures

#### Optimization of experimental conditions of Fe^3+^ ion Peroxidase-like activity to form the colored product

To evaluate the catalytic peroxidase-like properties of the Fe^3+^ ion, measurements of the blue product were performed at wavelength λmax 652 nm. The blue product was generated by the reaction between freshly prepared TMB and H_2_O_2_ in the presence of Fe^3+^ ion. At first, several parameters were optimized like Fe^3+^ ion concentration, pH, reaction time, TMB concentration and temperature. The preliminary ranges for Fe^3+^ ion concentration, pH, TMB concentration, reaction time, and temperature were optimized based on published work by Wu et al. [34]. In a typical experiment, 1 mL ferric chloride solution and 1 mL TMB solution at the optimized concentrations were added in 7 mL sodium acetate buffer solution of pH 4.2. Then 1 ml of 10 µM H_2_O_2_ solution was added. The resulting solution was kept in a temperature-controlled water bath at 30 °C for 15 min. The absorbance of the mixture was measured spectrophotometrically at a λmax 652 nm wavelength. The blank experiment was carried out under the same conditions without adding H_2_O_2_. The absorbance at 652 nm, represented as ΔA = A − A0, was used to quantify H_2_O_2_, where A and A0 are the absorbance in the presence and absence of H_2_O_2_, respectively. Optimization of the parameters was performed as described below. The percentage relative standard deviation (% RSD) values were calculated as follows: % RSD = (Standard Deviation/Mean) × 100.

##### Effect of Fe^3+^ ion concentration

To investigate the effect of changing Fe^3+^ ion concentration, the peroxidase-like activity of Fe^3+^ ion was measured when concentration of ferric chloride varied from 1.25 to 50 µM.

##### Effect of pH

Next, pH of sodium acetate buffer solution was studied between (4.0 to 5.0)

##### Effect of reaction time

Measurement of the blue color generated through peroxidase -like catalytic activity of Fe^3+^ ion is carried out after reaction time of 10, 15, 25, 35, and 45 min.

##### Effect of TMB concentration

Moreover, the effect of varying TMB concentration was examined from 0.005 to 0.02 mg mL^−1^.

##### Effect of temperature

To study the impact of the incubation temperature on the Fe^3+^ ion catalytic activity, the reaction mixture was incubated in a controlled water bath for 15 min. The temperature was varied from 20 to 40 °C.

#### Spectrophotometric determination of l-lactate using Peroxidase-like activity of Fe^3+^ ion

For l-lactate determination, a series of 50,75,100,150,175 and 200 µL of 1mM l-lactate standard stock solution were accurately transferred into a set of 20-mL test tubes. Then, 10 µL of 1.22 mg mL^−1^ LOx was added to each and completed with 50mM Tris HCl buffer (pH 8.5) to reach 1 mL. It was mixed and incubated at 37 °C water bath for 30 min. To the above mixture, 1 ml TMB solution (0.1 mg mL^−1^ TMB in sodium acetate buffer, pH 4.2), 1 ml of ferric chloride solution (250 µM) and 7 ml sodium acetate buffer, pH 4.2 were added to reach 10 mL final volume. After that, it was shaken well to mix the reagents. The mixture was incubated at 30 °C water bath for 15 min. After the specified time, the absorbance was recorded at λmax 652 nm with the spectrophotometer. The blank experiment was carried out under the same conditions without adding l-lactate. The absorbance at 652 nm, represented as ΔA = A − A0, was used to quantify l-lactate, where A and A0 are the absorbance in the presence and absence of l-lactate, respectively. Linear relationship relating the absorbance at λmax 652 nm to the corresponding concentrations of l-lactate was constructed and the respective regression equation was computed.

#### Spectrophotometric determination of l-lactate in presence of interferents and in artificial saliva

Artificial saliva was prepared as reported in literature [35], then spiked samples were utilized to determine the l-lactate in saliva. Accurate amounts of l-lactate were transferred into saliva samples to reach concentrations within the linearity range. Then, these samples were treated and tested through processes mentioned above. To evaluate the selectivity of the colorimetric l-lactate biosensor, 20 µM l-lactate was used in the presence of each interferent separately at concentrations that 20-folds of their salivary concentrations, such as 100 µM uric acid, 0.03 mM glucose, 0.16 mM pyruvate and 4 µM ascorbic acid. Furthermore, the phosphate ion as a potential interferent was studied by comparing the results obtained from artificial saliva samples prepared without any source of phosphate ion with those obtained from artificial saliva.

## Results and discussion

### Optimization of experimental conditions of Fe^3+^ ion peroxidase-like activity to form the colored product

**Scheme 1:**
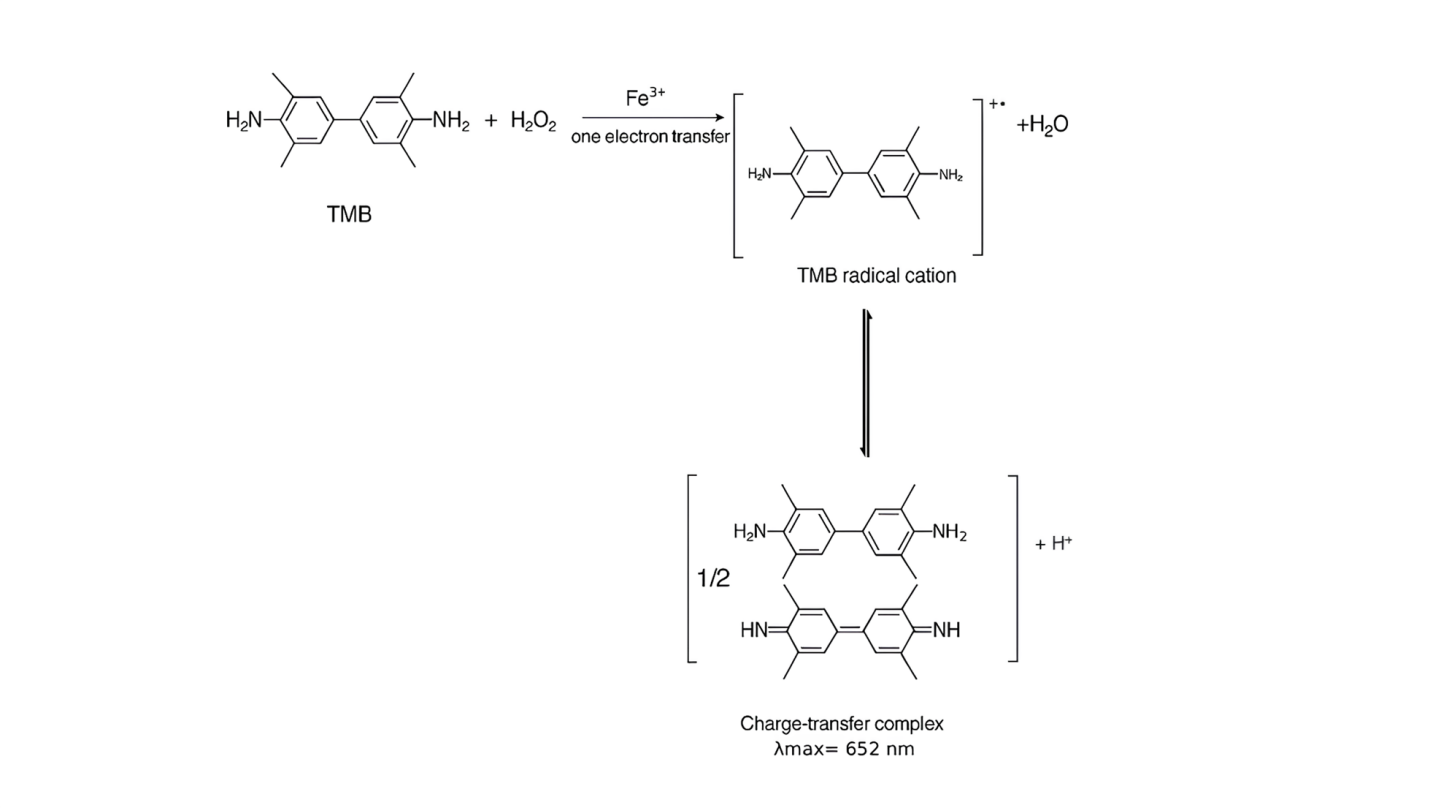
Reaction mechanism of TMB- H_2_O_2_ system in presence of Fe^3+^ ion

When H_2_O_2_ was incubated with TMB substrate solution in sodium acetate buffer at pH 4.2 in the presence of ferric chloride solution at the specified concentrations for 15 min at 30 °C, a blue colored product was obtained. There was a characteristic peak (Figure S1) appearing on the absorption spectrum of the test solution at λmax 652 nm due to the formation of the TMB charge-transfer complex [36]. The reaction mechanism was schematically shown in Scheme 1 [37, 38]. The observed peroxidase-like activity originates from the Fenton reaction of ferric/ferrous ions. The Fenton reaction involves interaction between Fe^2+^ and H_2_O_2,_ generates hydroxyl radicals that can oxidize TMB from a colorless to a blue compound [39]. Scheme S1 summarizes the mechanism of Fenton reaction [40]. Peroxidase-like activity of Fe^3+^ ion is reported to be higher than that of iron oxide magnetic nanoparticles. In addition fabrication of nanoparticles is complicated, energy- consuming and very costly to produce [34, 41]. In this work, ferric chloride which has peroxidase-like activity is used to design l-lactate biosensor rather than using iron oxide nanoparticles. The widely used TMB system was adopted due to its good stability [42].

$$\mathbf{L}{\mathrm{-}}\mathbf{l}\mathbf{a}\mathbf{c}\mathbf{t}\mathbf{a}\mathbf{t}\mathbf{e}\,+\,\mathbf{O}{\mathbf{2}}\xrightarrow{{{\mathbf{LOx}}}}{\mathbf{pyruvate}}\,+\,{{\mathbf{H}}_{\mathbf{2}}}{{\mathbf{O}}_{\mathbf{2}}}$$

$${{\mathbf{H}}_{\mathbf{2}}}{{\mathbf{O}}_{\mathbf{2}}}\,+\,{\mathbf{TMB}}\xrightarrow{{{\mathbf{F}}{{\mathbf{e}}^{3+}}}}{\mathbf{TM}}{{\mathbf{B}}_{{\mathbf{ox}}}}+{\text{ }}{{\mathbf{H}}_{\mathbf{2}}}{\mathbf{O}}$$

It is well-known that the peroxidase-like catalytic activity of Fe^3+^ ion towards the TMB–H_2_O_2_ system is dependent on pH, temperature, concentration of H_2_O_2_ and Fe^3+^ ion [34]. These parameters may significantly affect the Peroxidase -like activity of Fe^3+^ ion. Accordingly, the effect of ferric chloride concentration, pH, reaction time, TMB concentration and temperature to the peroxidase like activity of Fe^3+^ ion were studied and optimized at first. All of the optimization experiments were carried out in the acetic acid - sodium acetate buffer (pH 4.2).

#### Effect of Fe^3+^ ion concentration

Ferric ion concentration has a significant effect on its peroxidase -like catalytic activity. Experimental study on different concentrations of ferric chloride from 1.25 µM to 50 µM was performed. It is apparent that peroxidase -like catalytic activity increased in a strong magnitude from 1.25 µM till 12.5 µM of ferric chloride, then no increase in the peroxidase-like activity was observed. As shown in Figure S2, optimum color was obtained with concentration of 25 µM ferric chloride solution. Reproducibility (RSD%) was found to be 1.79%.

#### Effect of pH

**Fig. 1:**
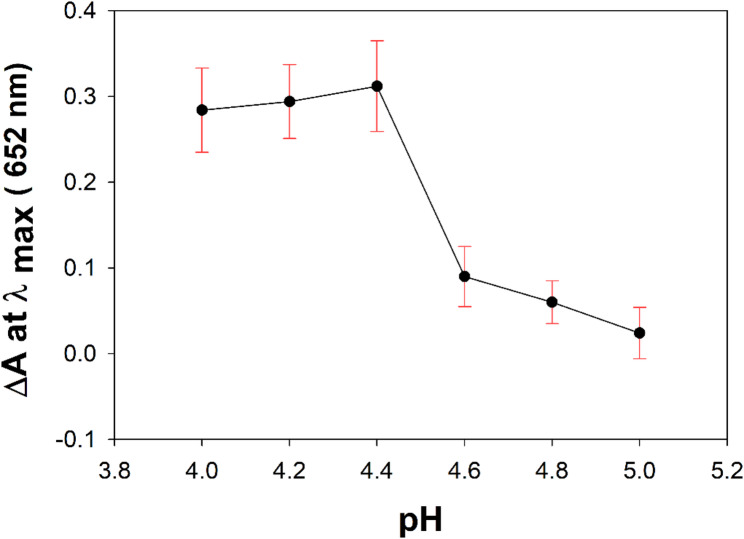
pH-dependent peroxidase-like activity of Fe^3+^ ion towards the TMB/H_2_O_2_ system at λmax 652 nm. Experimental conditions: [TMB] = 0.01 mg mL^−1^; [H_2_O_2_] = 10 µM; [Fe^3+^] = 25 µM; temperature of 30 °C; time = 15 min

The pH effect of acetate buffer on the peroxidase -like catalytic activity of Fe^3+^ ion was optimized within the capacity of acetate buffer in the range of pH (4.0 to 5.0). It was found that the catalytic activity of Fe^3+^ ion increased with increasing the pH up to 4.4, but subsequently declined with increasing pH values (Fig. 1) [43–46]. RSD% was determined and found to be 3.97%.

#### Effect of reaction time

The reaction time was investigated from 10 to 45 min as presented in Figure S**3**. The optical density of the blue color generated through peroxidase -like catalytic activity of Fe^3+^ ion was found to reach a maximum intensity within 15 min. Longer reaction times led to the precipitation of oxidized TMB [47]. The estimated RSD% results for reaction time was 0.15%.

#### Effect of TMB concentration

The optimum concentration of TMB was investigated in the range of 0.005 to 0.02 mg mL^−1^. It was observed that, higher optical density of blue color was obtained with higher concentration of TMB (Figure S4). The optimum concentration of TMB which achieved good linearity of the method for the biosensor analytical validation is 0.01 mg mL^−1^. RSD % was calculated and found to be 0.73%.

#### Effect of temperature

**Fig. 2:**
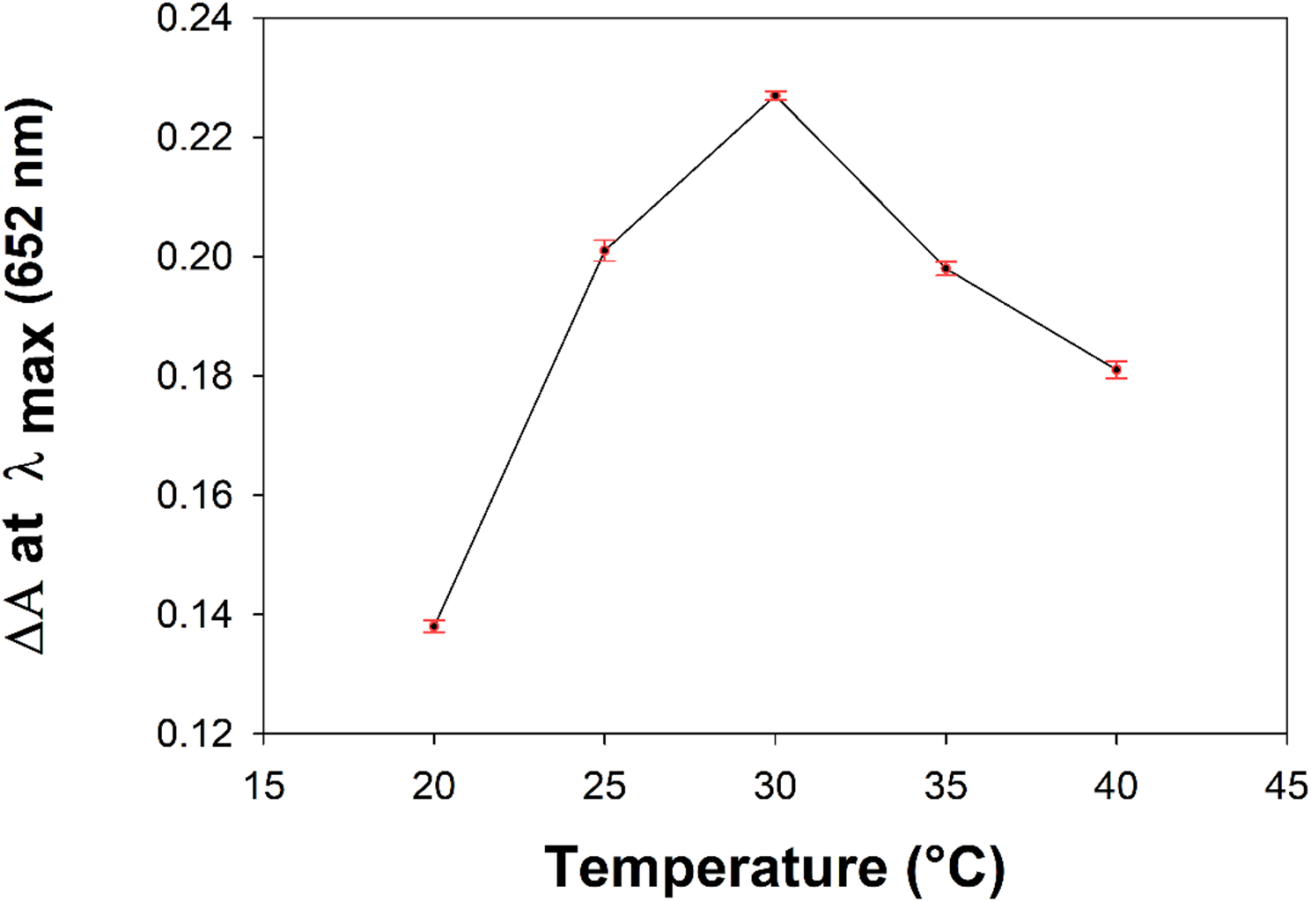
Temperature-dependent peroxidase-like activity of Fe^3+^ ion towards the TMB/H_2_O_2_ system at λmax 652 nm. Experimental conditions: [Fe^3+^] = 25 µM; [H_2_O_2_] = 10 µM; [TMB] = 0.01 mg mL^−1^; time = 15 min; sodium acetate buffer solution of pH 4.2

The impact of the incubation temperature on the Fe^3+^ ion catalytic activity is shown in Fig. 2. This activity was studied upon varying the incubation temperature from 20 to 40 °C. The optimum peroxidase -like catalytic activity of Fe^3+^ ion was found to be at 30 °C. Therefore, an incubation temperature of 30 °C was selected to be controlled throughout the method. Reproducibility of temperature optimization experiments was expressed as %RSD and found to be 0.183%.

### Spectrophotometric determination of l-lactate using PEROXIDASE-like activity of Fe^3+^ ion

**Fig. 3:**
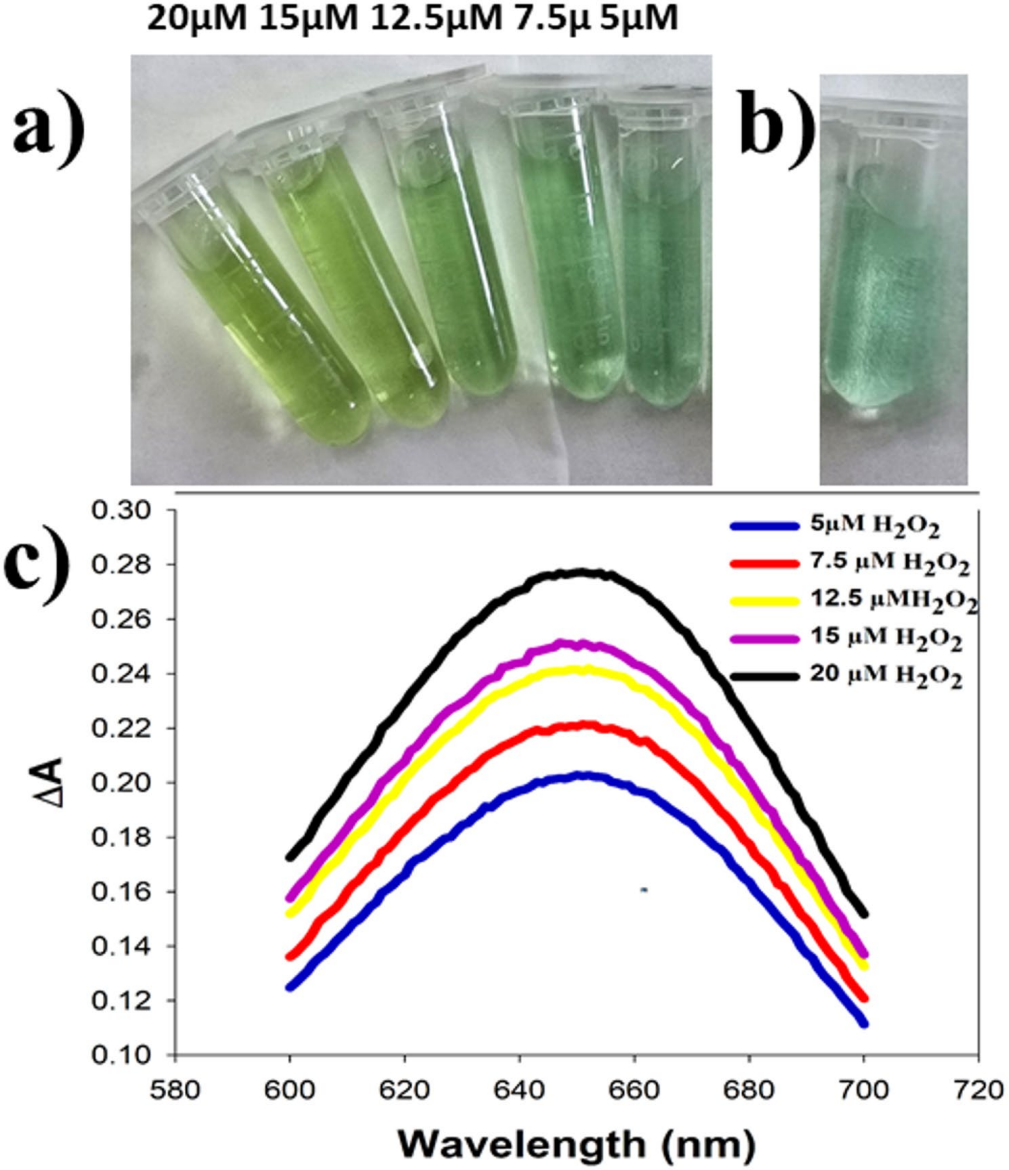
**a** Photograph of Color of different H_2_O_2_ concentration in the linear range (5, 7.5, 12.5, 15 and 20) µM from right to left, **b** Photograph of blank experiment without H_2_O_2_ and **c** UV-vis spectra of different H_2_O_2_ concentration in the linear range (5, 7.5, 12.5, 15 and 20) µM

**Fig. 4:**
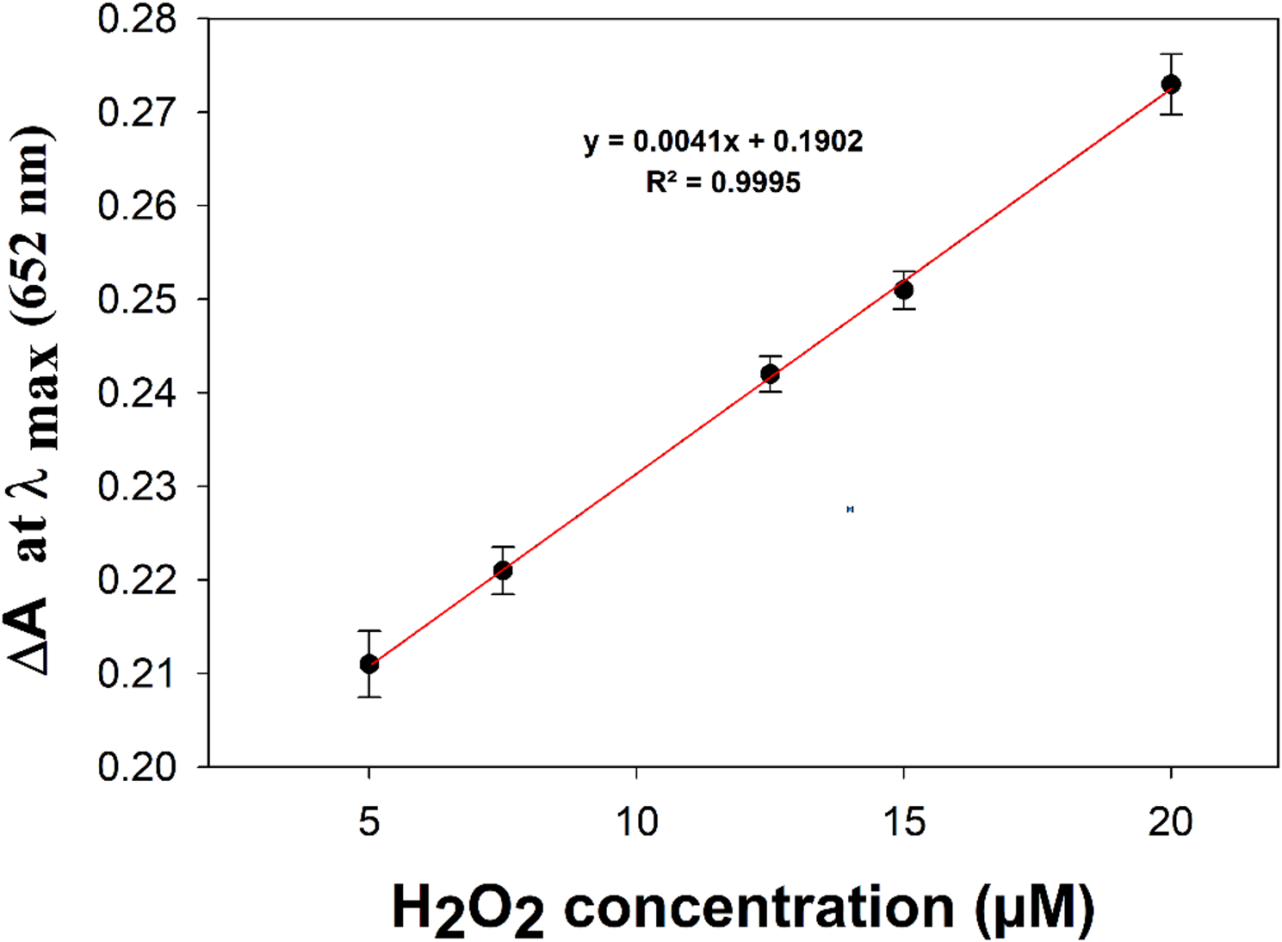
Linearity of absorbance to the corresponding H_2_O_2_ concentration at λ_max_ 652 nm (error bars represent standard deviation of mean [50], *n* = 3)

Based on the peroxidase -like catalytic activity of Fe^3+^ ion, ferric chloride and LOx were used as a lactate biosensor. For this procedure, it was expected that l-lactate oxidation reaction by LOx leads to the formation of H_2_O_2_, which reacts quantitatively with TMB in the presence of Fe^3+^ ion to generate a colored product that could be measured spectrophotometrically. Based on the reaction parameters for oxidizing l-lactate from previous studies, lactate oxidase enzyme was utilized to convert l-lactate to pyruvate and H_2_O_2_ [48, 49]. The released H_2_O_2_ and thus lactate concentration was determined using peroxidase-like activity of Fe^3+^ ion under the optimized assay conditions. Table 1 shows the optimized conditions which subsequently were adopted. UV-vis spectra of linear calibration curve of H_2_O_2_ along with photograph of color changes is presented in Fig. 3. The calibration curve of the absorbance at λmax 652 nm versus H_2_O_2_ concentration (Fig. 4) showed linearity in the range of 5 µM to 20 µM with a limit of detection of 0.455 µM. The correlation between the concentration and absorbance value fitted the equation of ΔA = 0.0041 C + 0.1902 where ΔA is the absorbance at λmax 652 nm, C is the concentration of H_2_O_2_ in µM. Excellent agreement between the data and the regression model for the calibration curve has been indicated by the coefficient of determination (R^2^) of 0.9995. Linearity range, regression equation, correlation coefficient, limit of quantification and limit of detection are all summarized in Table 2.

**Table 1:** Optimized conditions for the established spectrophotometric method for l-lactate determination

Reaction conditions for oxidation of l-lactate
LOx :0.5 U mL^−1^ Reaction pH: 8.5 Buffer solution: 50 mM Tris HCl buffer Reaction time: 30 min Reaction temperature: 37 °C
Incubation conditions
TMB concentration: 0.01 mg mL^−1^ Ferric chloride concentration: 25 µM Buffer solution: 0.2 M acetate buffer, pH:4.2 Incubation time: 15 min Incubation temperature: 30 °C

**Table 2:** Validation parameters (regression and validation data) of the proposed biosensor using hydrogen peroxide, the lox product

Parameter	
Linearity range	5–20 µM
Regression equation	ΔA = 0.0041 C + 0.1902
Slope	0.0041
Intercept	0.1902
Correlation coefficient	0.9997
Accuracy (mean ± SD)	100.46 ± 0.514
LOD^a^	0.455 µM
LOQ^b^	1.380 µM
Precision (%RSD)	
Intraday precision—repeatability	1.788
Interday precision—intermediate precision	1.970

^a^ LOD = 3.3* σ/S

^b^ LOQ = 10* σ/S

where σ is the calculated SD of residuals of the regression line, and S is the slope of the obtained calibration curve

Independent testing using the same procedure, in the same laboratory and equipment, and under the same conditions determined the proposed method’s precision. Three different concentrations from the analytical curve (15 µM, 19 µM and 20 µM) were repeated at various times both on the same day (intraday precision) and on three consecutive days (interday precision) in order to perform the repeatability experiments. Three experiments were carried out and the absorbance values were recorded for each concentration. The precision was expressed using the relative standard deviations (%RSD). The %RSD values obtained for intraday and interday precision were 1.788% and 1.970 respectively. These findings, which assured that the proposed sensor response is reproducible and that l-lactate could be determined using Fe^3+^ ion with colorimetric detection, could potentially be considered satisfactory.

**Fig. 5:**
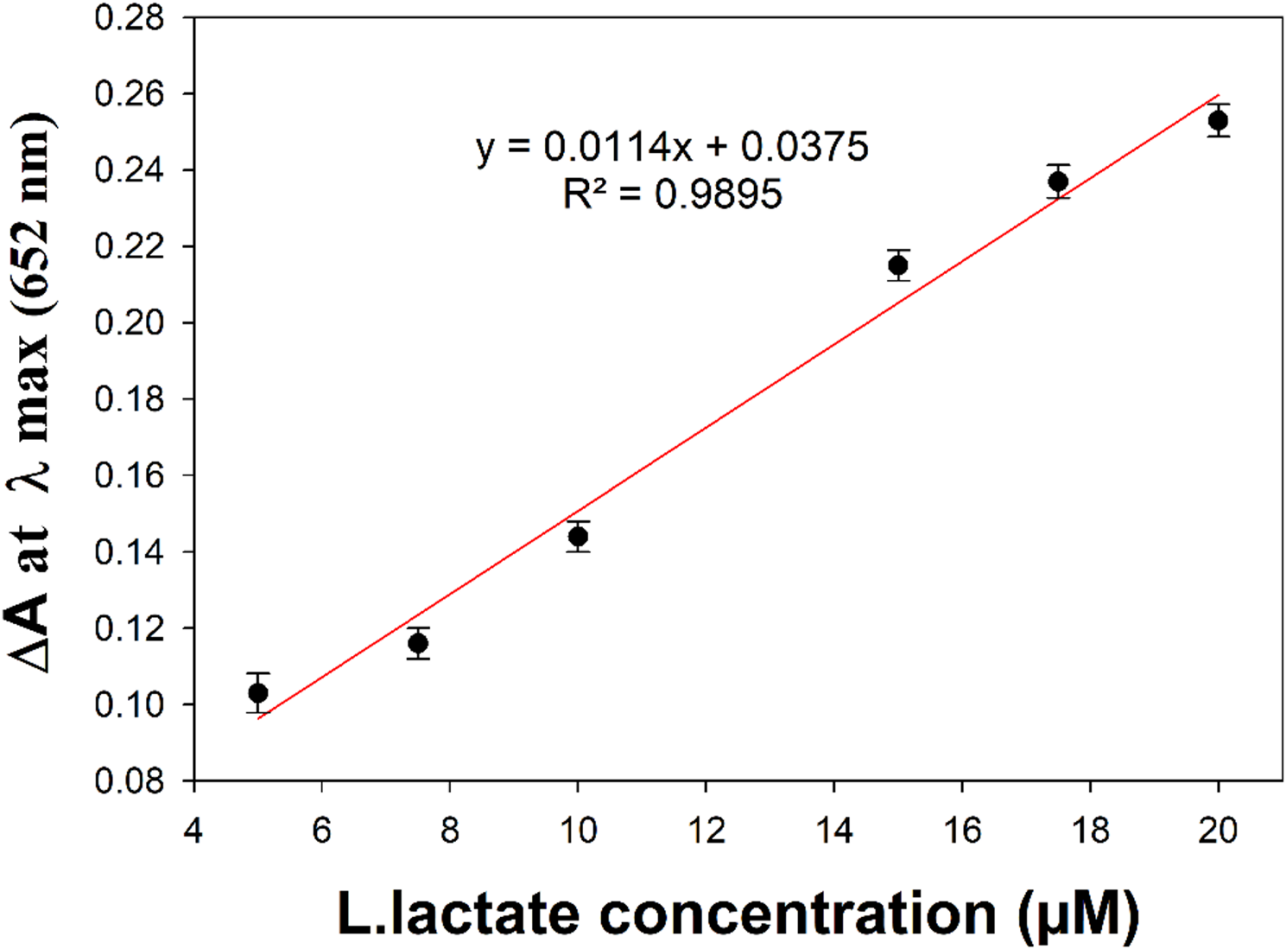
Linearity of absorbance to the corresponding l-lactate solution concentration at λ_max_ 652 nm. (Error bars represent standard deviation of mean [50], *n* = 3)

Because H_2_O_2_ is the main product of the lactate oxidase (LOx)-catalyzed reaction, therefore, when combined with lactate oxidase (LOx), the proposed method could be used for the determination of l-lactate. UV-vis spectra of linear calibration curve of l-lactate along with photograph of color changes is presented in Figure S5. The calibration curve of the absorbance at λmax 652 nm versus l-lactate concentration is presented in Fig. 5. The regression equation is ΔA = 0.0114 C + 0.0375, R^2^ = 0.9895 where ΔA is the absorbance at λmax 652 nm, C is the concentration of l-lactate µM in and R^2^ is the coefficient of determination. Table 3 summarizes all the regression data regarding linearity range, regression equation, correlation coefficient, limit of quantification and limit of detection.

**Table 3:** Regression data of the proposed biosensor for spectrophotometric determination of l-lactate

Parameter	
Linearity range	5–20 µM
Regression equation	ΔA = 0.0114 C + 0.0375
Slope	0.0114
Intercept	0.0375
Correlation coefficient	0.995
LOD^a^	1.278 µM
LOQ^b^	3.871 µM

^a^LOD = 3.3* σ/S

^b^LOQ = 10* σ/S

where σ is the calculated SD of residuals of the regression line, and S is the slope of the obtained calibration curve

### Spectrophotometric determination of l-lactate in presence of interferents and in artificial saliva

**Fig. 6:**
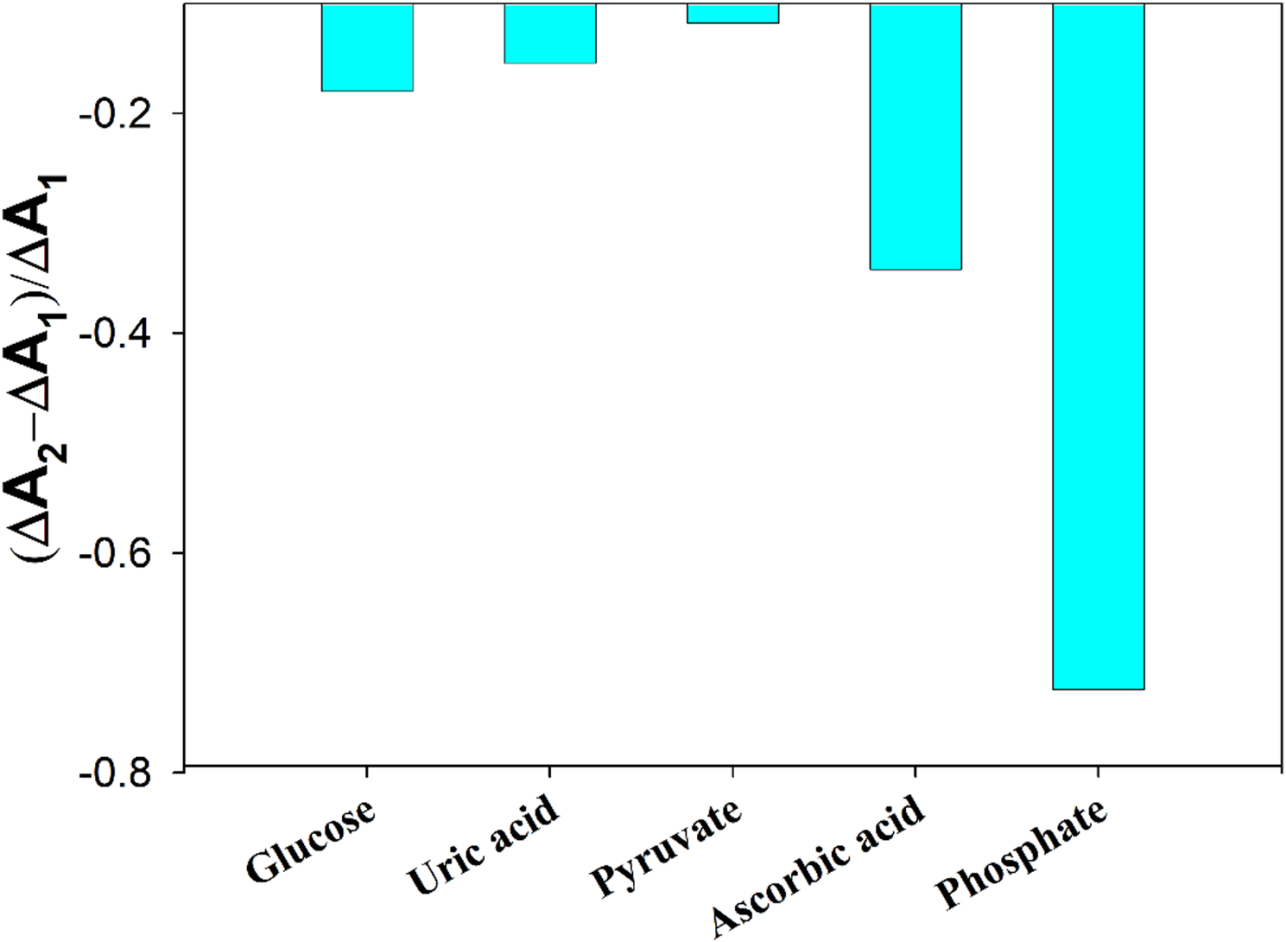
Selectivity study of LOx/ FeCl_3_/TMB in the presence of 20 µM lactate and interferents at 652 nm (pH 4.2)

To evaluate the selectivity of the l-lactate biosensor, the proposed method was performed in the presence of potential interferents such as glucose, uric acid, pyruvate and ascorbic acid. The specificity of the enzymatic systems for analyte determination is well established [51]. The proposed method is mainly dependent on the selectivity of lactate oxidase enzyme to the substrate. The ratio of absorbance change ((ΔA_2_– ΔA_1_)/ ΔA_1_ where ΔA_2_ and ΔA_1_ are the absorbance of l-lactate in the presence and the absence of interferent, respectively) of 20 µM l-lactate was calculated. The obtained results express the contribution of about 20-fold of potential interferents in the real saliva matrix at ΔA of the l-lactate biosensor. The results are presented in Fig. 6, the ratio of ΔA change was found to be − 0.18, − 0.15, − 0.12 and − 0.34 for glucose, uric acid, pyruvate and ascorbic acid respectively. Such interferents even at such high concentration (~ 20-fold normal physiological range) expressed negligible impact on l-lactate measurement that could confirm the promising anti-interference capability of the proposed biosensor. The interference of ascorbic acid may originate from the reduction of Oxidized TMB [52] or ferric chloride [53]. Phosphate ion may be considered as a potential interfering component in artificial saliva due to its inhibitory effect on peroxidase-mimetic activity of ferric ions as reported in the literature [54]. Hence, artificial saliva has been prepared with the omission of phosphate ions. The ratio of ΔA change in l-lactate quantification due to phosphate ion was found to be − 0.72. To overcome the matrix effect as a very common challenge in biological analysis, several methods could be utilized. Those methods include sample preparation methods and calibration correction methods like matrix-matched calibration, internal standard and standard addition [55]. Therefore, to eliminate interference from phosphate ions, a matrix-matched calibration curve method has been adopted to quantify the unknown saliva samples.

To evaluate the feasibility of our study for analysis of l-lactate in biological samples, our proposed sensor was used to determine l-lactate concentration in artificial saliva. As for the assay of l-lactate, the experiment was performed by spiking different amounts of l-lactate into some artificial saliva samples and then the mixed samples were determined as explained in the experimental section. After the specified time is completed, the absorbance of the produced color was recorded. The content of l-lactate in different artificial saliva samples was computed according to the linear equation obtained from matrix -matched calibration curve (ΔA = 0.0053 C – 0.0177 where ΔA is the absorbance at λmax 652 nm, C is the concentration of l-lactate in µM). Results are listed in Table 4. According to ICH bioanalytical method validation guidelines, the mean accuracy should be within ± 15–20% of the nominal concentration [56]. As can be concluded, the accuracy results of the proposed method for detecting l-lactate in saliva were satisfactory and within the acceptance criteria, thus ferric ion was promising as peroxidase mimetics for potential application of l-lactate determination clinically. A comparison between the analytical performance of the proposed method and previously reported methods for determination of l-lactate is given in Table S1 in supporting information.

**Table 4:** Spectrophotometric determination of l-lactate in the artificial saliva samples using the proposed method

Sample	Lactate added into artificial saliva (µM)	Found^*^ (µM)	Recovery %
Sample I	6	5.792	96.53
Sample II	7.5	7.717	102.89
Sample III	12.5	12.318	98.54
Sample IV	15	15.727	104.85

* Values are the average of three independent analysis of the same sample (n ꞊ 3)

## Conclusion

A colorimetric biosensor was developed for quantification of l-lactate biomarker. This was carried out by employing lactate oxidase enzyme to oxidize l-lactate into pyruvate and H_2_O_2_ after 30 min incubation at 37 °C water bath. The liberated H_2_O_2_ reacts with Fe^3+^ ion which has inherent peroxidase-mimetic activity instead of using peroxidase enzyme at 30 °C water bath for 15 min. The developed biosensor provides an accurate, precise, sensitive, and cost-effective approach to determine l-lactate. The analytical performance was evaluated and showed adequate precision and accuracy. The assay has a linear range of 5 µM–20 µM of l-lactate, detection limit of 1.278 µM and Limit of quantitation of 3.871 µM. This sensor enables the determination of l-lactate in artificial saliva samples with recoveries within the acceptance criteria. The developed method has ensured selectivity being based on lactate oxidase enzyme. The proposed method eliminates the need for any hazardous solvents and sophisticated instrumentation.

## Supplementary Information

Below is the link to the electronic supplementary material.

Supplementary Material 1.

## Data Availability

All data generated or analysed during this study are included in this published article and its supplementary information file.

## Abbreviations

HRP: Horseradish peroxidase
Lox: Lactate oxidase enzyme
OPD: *o*-Phenylenediamine dihydrochloride
POD: Peroxidase
TMB: 3,3′,5,5′-Tetramethylbenzidine
