# Supplementary material for: Development of a biosensor for spectrophotometric determination of l-lactate in artificial saliva
Source: BMC Chem. 2026 Jan 30;20(1):39. doi: 10.1186/s13065-025-01718-5 (PMC12930734; doi:10.1186/s13065-025-01718-5)
Supplement: Supplementary file 1 — Supplementary Material 1. [file 13065_2025_1718_MOESM1_ESM.docx]

Supporting Information

for

Development of a Biosensor for Spectrophotometric Determination of L-lactate in Artificial Saliva

Rehab E. Bayoumy^*^, Nariman A. El-Ragehy, Nagiba Y. Hassan, Amr M. Mahmoud^*^

Pharmaceutical Analytical Chemistry Department, Faculty of Pharmacy, Cairo University, Kasr-El-Aini, 11562 Cairo, Egypt

^*^Corresponding author email: [rehab.essam@pharma.cu.edu.eg](mailto:rehab.essam@pharma.cu.edu.eg) , [amr.bekhet@pharma.cu.edu.eg](mailto:amr.bekhet@pharma.cu.edu.eg)


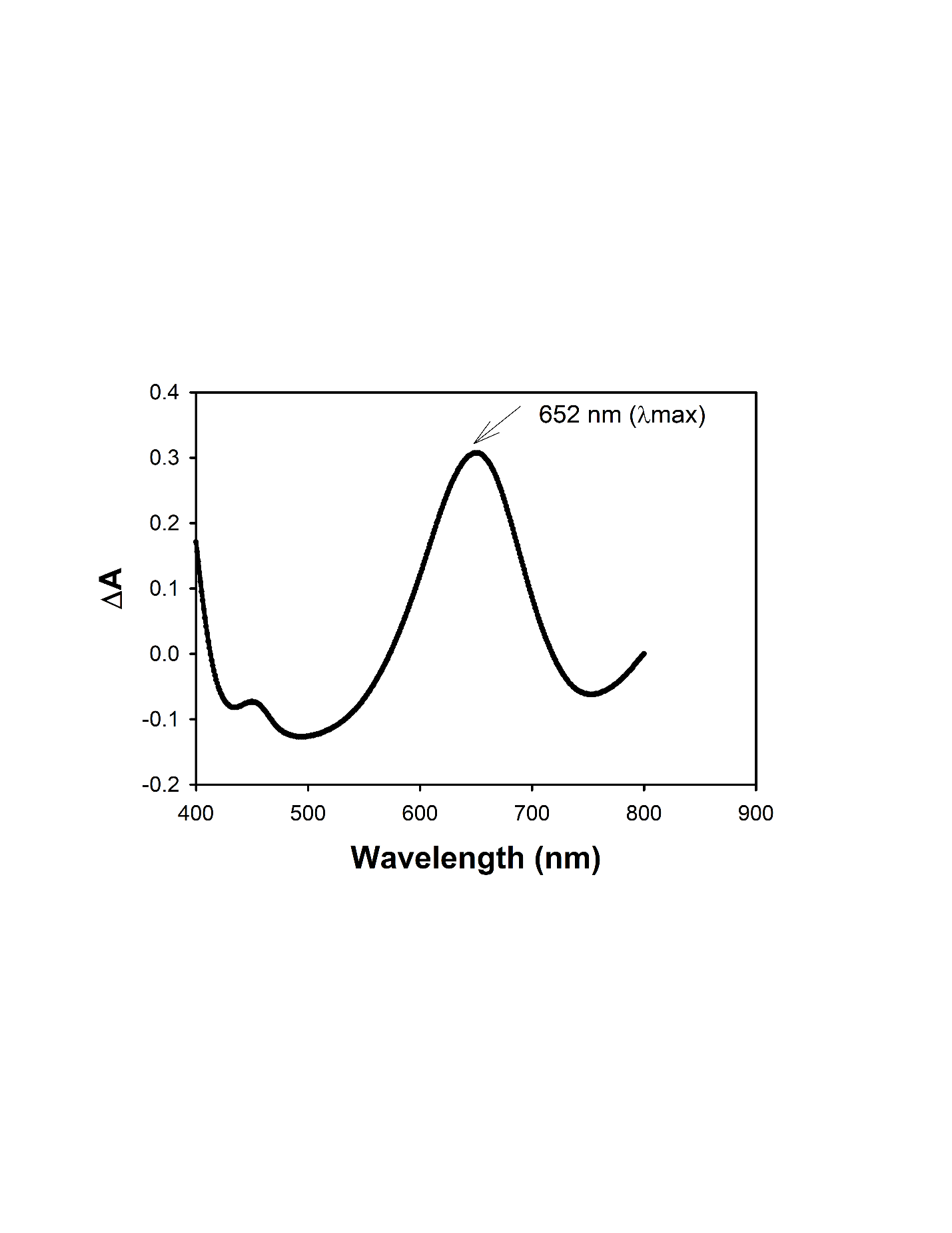


**Figure S1**: Absorption spectrum of the reaction mixture of 1 ml 0.1 mg. mL^−1^ TMB with 1 ml 250 μM FeCl_3_ and 10 μM H_2_O_2_ incubating in sodium acetate buffer (pH 4.2) at 30 °C water bath for 15 min.

**Figure S2**: Fe^3+^ ion concentration-dependent peroxidase-like activity towards the TMB/H_2_O_2_ system at λmax 652 nm. Experimental conditions: [TMB] = 0.01 mg. mL^−1^; [H_2_O_2_] = 10 μM; temperature of 30 ^o^C; time =15 min; sodium acetate buffer solution of pH 4.2.

*
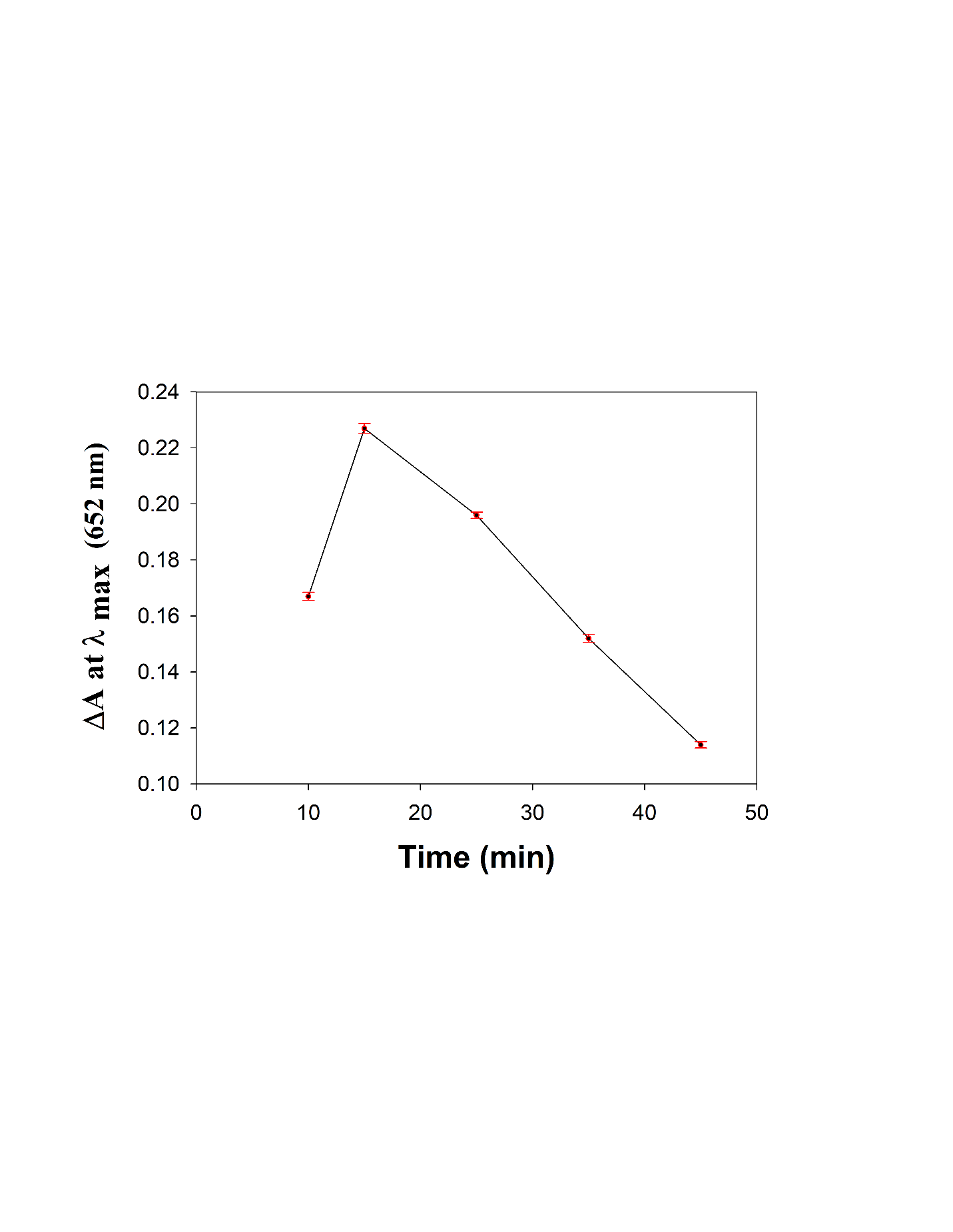
*

**Figure S3**: Time-dependent absorbance changes at λmax 652 nm of the Fe^3+^/TMB/H_2_O_2_ system. Experimental conditions: [TMB] = 0.01 mg. mL^−1^; [H_2_O_2_] = 10 μM; [Fe^3+^] = 25 μM; temperature of 30 ^o^C; sodium acetate buffer solution of pH 4.2.


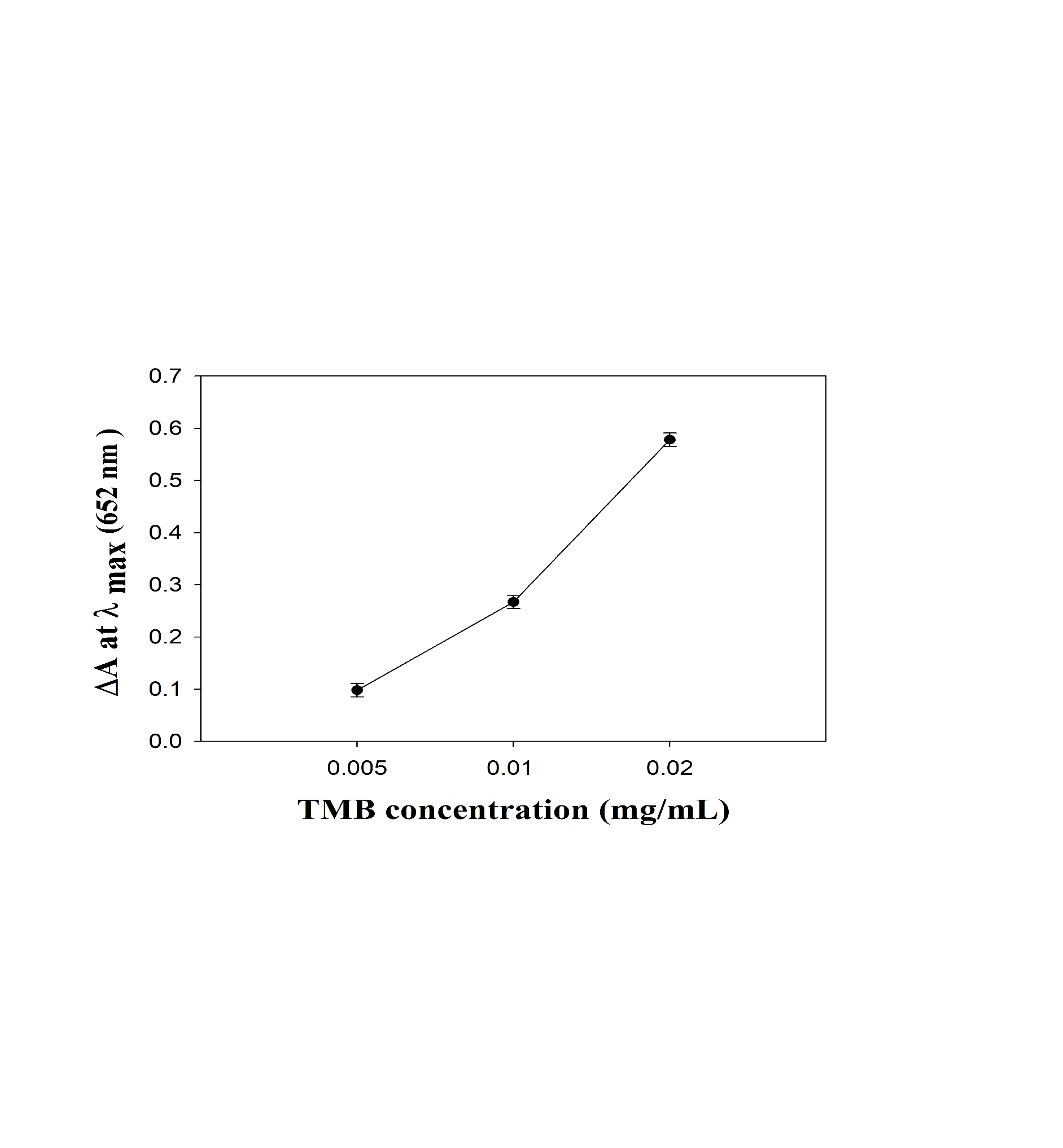


**Figure S4**: TMB concentration-dependent towards the Fe^3+/^TMB/H_2_O_2_ system at λmax 652 nm. Experimental conditions: [Fe^3+]^ = 25 μM; [H_2_O_2_] = 10 μM; temperature of 30 ^o^C; time =15 min; sodium acetate buffer solution of pH 4.2


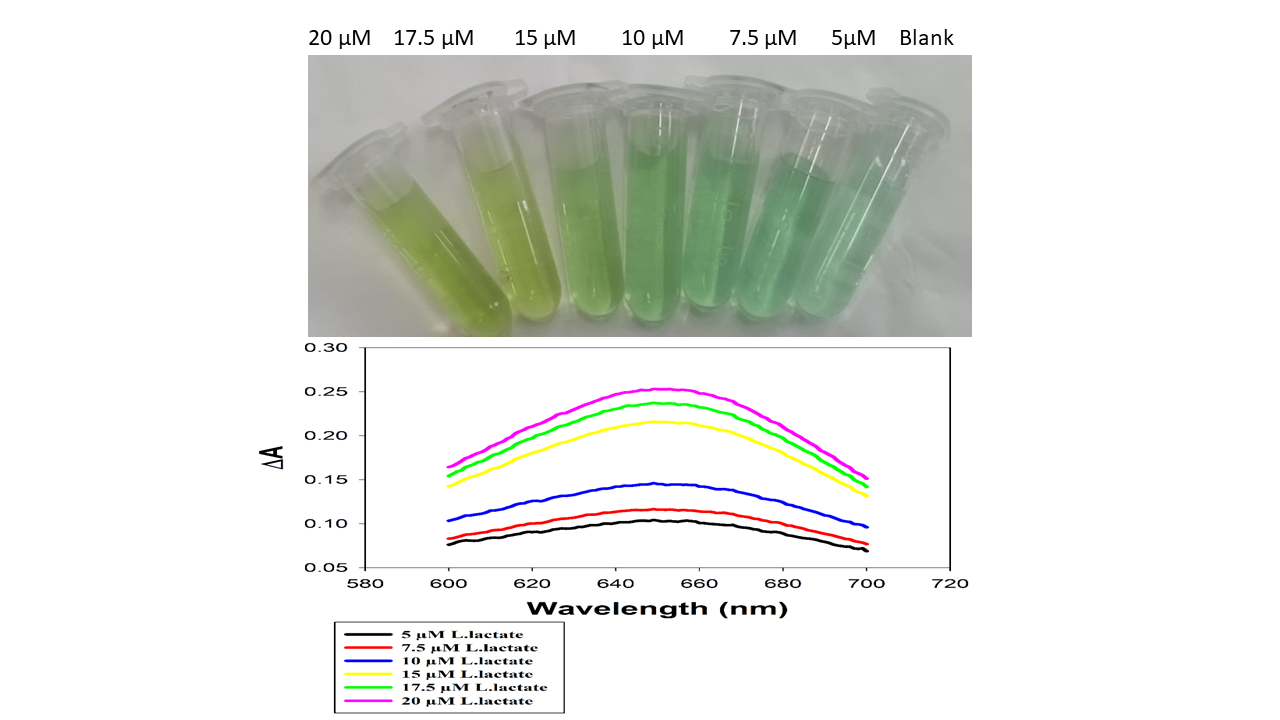


**Figure S5**: UV-vis spectra of different L-lactate concentrations in the linear range of

(5-20) μM


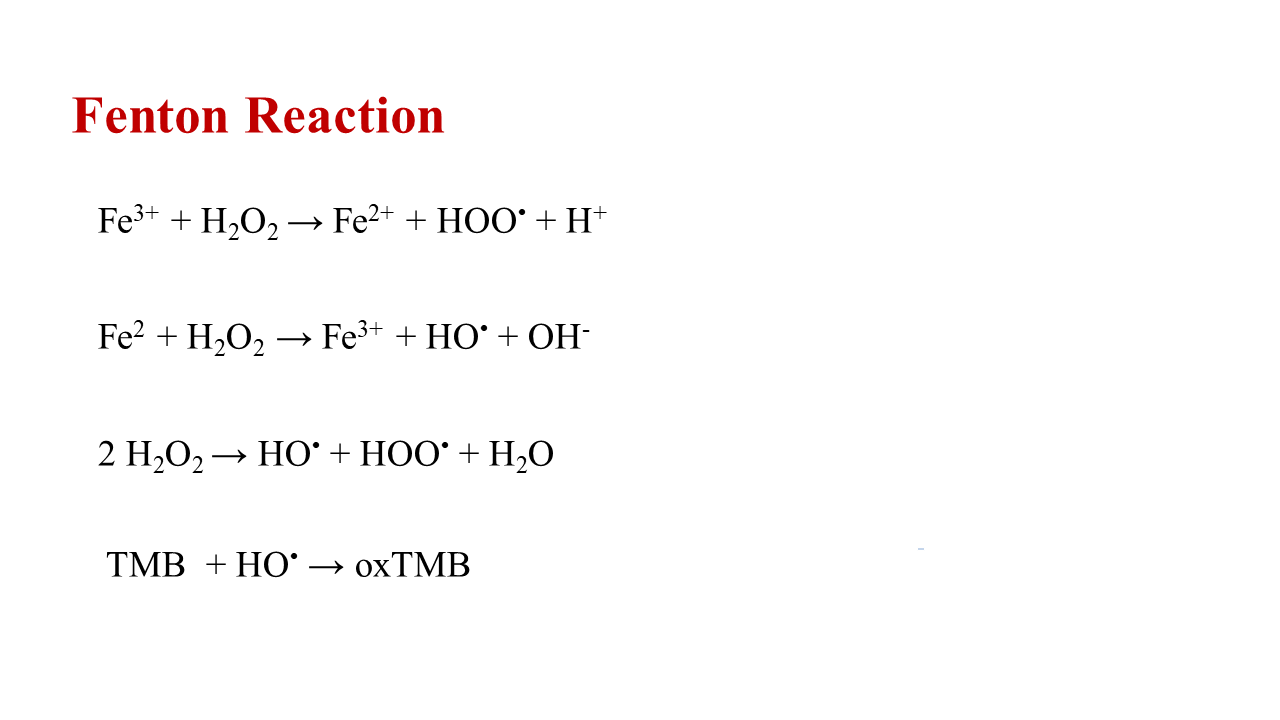


Scheme S1: Mechanism of Fenton reaction generating Hydroxyl radical

**Table S1: An overview of analytical Performance of different methods for determination of L-Lactate**

| **Sensor** | **Method** | **Linear range** | **LOD** | Sensitivity | Application | Ref |
| --- | --- | --- | --- | --- | --- | --- |
| NADH/LDH/NanoCeO2/GCE | Cyclic Voltammetry | 200-2000 μM | 50 μM | 571.19 | - | (1) |
| LOx/DNPs/Au | Cyclic Voltammetry | 0.05-0.7 mM | 15 μM | 4 | - | (2) |
| SPE-PB-LOx biosensor | Chronoamperometry | 25-250 μM | 10 μM | - | Saliva and Blood samples | (3) |
| Au-Ag/C NC | Colorimetric | 0.1-22 μM & 22- 220 μM | 0.03 μM | - | Serum samples. | (4) |
| PtNPs coated 3D-printed multi-well plate | Colorimetric | 2.5-100 μM | 0.6 μM | 0.00166 | Urine , plasma and rat brain microdialysate samples. | (5) |
| C_3_N_4_ nanosheet-supported PB NPs | Colorimetric | 5- 100 μM | 2.2 μM | 3.30 | Serum samples. | (6) |
| LOx + HRP /CDs on 3D-μPADs | Fluorimetric | 2.5-20 μM | 0.814 μM | -10.3× 10^3^ | Saliva samples | (7) |
| γ-/α- Fe_2_O_3_ and γ-/α- Fe_2_O_3_/ZnO NPs | Colorimetric | 50 - 1000 μM | 4.3 μM and 9.4 μM | - | Serum samples. | (8) |
| Polyaniline NPs -Based Sensor | Colorimetric | 1 -100 mM. | 1 mM | - | Sweat samples | (9) |
| LOD-GO-ZnO modified  SPCE | Chronoamperometry | 15-1250 μM | 9 μM | 3.308 | Saliva samples | (10) |
| FeCl_3_- TMB LOx biosensor | Colorimetric | 5-20 μM | 1.278 μM | 0.0114 | Saliva samples | This work |

**References:**

1. Nesakumar N, Sethuraman S, Krishnan UM, Rayappan JBB. Fabrication of lactate biosensor based on lactate dehydrogenase immobilized on cerium oxide nanoparticles. Journal of Colloid and Interface Science. 2013;410:158-64.

2. Briones M, Casero E, Petit-Domínguez MD, Ruiz MA, Parra-Alfambra AM, Pariente F, et al. Diamond nanoparticles based biosensors for efficient glucose and lactate determination. Biosensors and Bioelectronics. 2015;68:521-8.

3. Petropoulos K, Piermarini S, Bernardini S, Palleschi G, Moscone D. Development of a disposable biosensor for lactate monitoring in saliva. Sensors and Actuators B: Chemical. 2016;237:8-15.

4. Zhang L, Hou W, Lu Q, Liu M, Chen C, Zhang Y, et al. Colorimetric detection of hydrogen peroxide and lactate based on the etching of the carbon based Au-Ag bimetallic nanocomposite synthesized by carbon dots as the reductant and stabilizer. Analytica Chimica Acta. 2016;947:23-31.

5. Su C-K, Li T-W, Sun Y-C. Peroxidase-mimicking PtNP-coated, 3D-printed multi-well plate for rapid determination of glucose and lactate in clinical samples. Sensors and Actuators B: Chemical,2018;269:46-53.

6. Zhou D, Wang C, Luo J, Yang M. C 3 N 4 nanosheet-supported Prussian Blue nanoparticles as a peroxidase mimic: colorimetric enzymatic determination of lactate. Microchimica Acta. 2019;186:1-8.

7. Rossini EL, Milani MI, Lima LS, Pezza HR. Paper microfluidic device using carbon dots to detect glucose and lactate in saliva samples. Spectrochimica Acta Part A: Molecular and Biomolecular Spectroscopy,2021;248:119285.

8. Escalona-Villalpando RA, Viveros-Palma K, Espinosa-Lagunes FI, Rodríguez-Morales JA, Arriaga LG, Macazo FC, et al. Comparative Colorimetric Sensor Based on Bi-Phase &gamma;-/&alpha;-Fe2O3 and &gamma;-/&alpha;-Fe_2_O_3_/ZnO Nanoparticles for Lactate Detection. Biosensors 2022;12(11):1025.

9. Kim HJ, Park I, Pack SP, Lee G, Hong Y. Colorimetric Sensing of Lactate in Human Sweat Using Polyaniline Nanoparticles-Based Sensor Platform and Colorimeter. Biosensors. 2022;12(4):248.

10. Han J, Shaohui J. Fabrication of a novel sensor for lactate screening in saliva samples before and after exercise in athletes. Alexandria Engineering Journal. 2024;92:171-5.
